# Assessment of UV radiation emitted by linear fluorescent lamps in Iran

**DOI:** 10.1016/j.mex.2019.02.031

**Published:** 2019-02-28

**Authors:** Mohammad Hadi Dehghani, Azam Bazargani, Mohammad Mirdoraghi

**Affiliations:** a Department of Environmental Health Engineering, School of Public Health, Tehran University of Medical Sciences, Tehran, Iran; b Institute for Environmental Research, Center for Solid Waste Research, Tehran University of Medical Sciences, Tehran, Iran; c Department of Radiology and Radiotherapy Technology, School of Allied Health Sciences, Tehran University of Medical Sciences, Tehran, Iran

**Keywords:** Measurement of UV radiation emitted by linear fluorescent lamps, Linear fluorescent lamps, Ultraviolet radiation, UVA, UVC

## Abstract

The aim of the study was to evaluate ultraviolet (UV) radiation emitted from linear fluorescent lamps widely used in Iran. This study was conducted on 40 linear fluorescent lamps (15 different brands) with different wattages. The UV radiation was measured at 2, 5, 20, 50, 150 cm for one, 5, 30, 60 min in 2 spectral values i.e. UVC (100 ≤ wavelength ≤ 280) and UVA (300 ≤ wavelength ≤ 400). Data analysis was carried out by SPSS version 18. There is a significant relationship between measured values at 2, 5, 20, 50, 150 cm with measured values of UVA at 2, 5, 20, 50,150 cm (P < 0.05). There was no significant relationship at other measured amounts (P > 0.05). The results showed that the intensity of UVA emitted from the lamps was less than the permissible limit at all distances. However, this value is higher than the permissible limit for UVC at 150 cm.

## Graphical abstract

**Figure:**
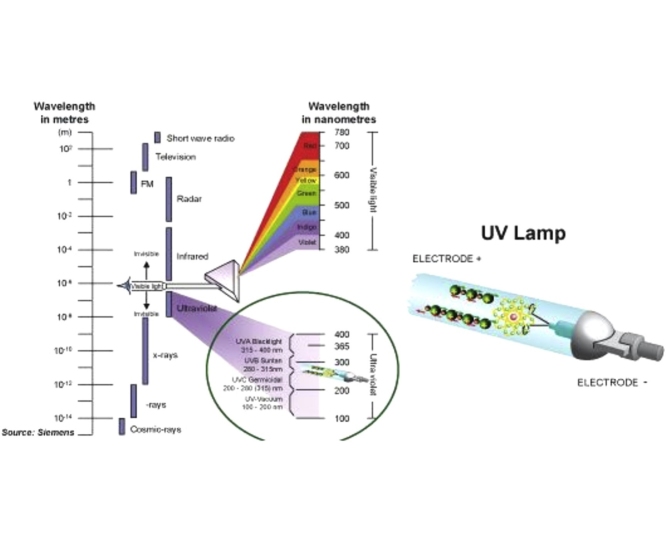

## Specifications Table

**Table:** 

**Subject Area:**	Environmental Science
**More specific subject area:**	Measurement of UV radiation
**Method name:**	Measurement of UV radiation emitted by linear fluorescent lamps.
**Name and reference of original method:**	UV Radiation Emitted by Linear Fluorescent Lamps in Iran
**Resource availability:**	data

**Table:** 

**Protocol data:**
• The data of UV measurements showed that the amount of UVA was less than the daily limit at all distances. • The measured values of UVC are higher than the permissible limit at 150 cm. • Increasing the number of fluorescent lamp coatings and designing an appropriate bubble can significantly reduce the amount of UV radiation emitted by linear fluorescent lamps.

## Description of protocol

Table 1 shows the results of univariate analysis of variance (ANOVA) of UV measurements at 2, 5, 20, 50, and 150 cm. Table 2 demonstrates the mean based on 2 parameters of distance and time.

**Table 1:** Comparison of mean UVA according to the distance from the linear fluorescent lamps.

(I) Distance (m)	(J) Distance (m)	Mean Difference (I-J)	Std. Error	Sig.	95% Confidence Interval		
					Lower Bound	Upper Bound	
2	5	0.10812	0.011386	0.000	0.07222		0.14403
	20	0.24100	0.011386	0.000	0.20509		0.27691
	50	0.28312	0.011386	0.000	0.24722		0.31903
	150	0.29312	0.011386	0.000	0.25722		0.32903
5	2	−0.10812	0.011386	0.000	−0.14403		−0.07222
	20	0.13288	0.011386	0.000	0.09697		0.16878
	50	0.17500	0.011386	0.000	0.13909		0.21091
	150	0.18500	0.011386	0.000	0.14909		0.22091
20	2	−0.24100	0.011386	0.000	−0.27691		−0.20509
	5	−0.13288	0.011386	0.000	−0.16878		−0.09697
	50	0.04213	0.011386	0.014	0.00622		0.07803
	150	0.05213	0.011386	0.002	0.01622		0.08803
50	2	−0.28312	0.011386	0.000	−0.31903		−0.24722
	5	−0.17500	0.011386	0.000	−0.21091		−0.13909
	20	−0.4213	0.011386	0.014	−0.07803		−0.00622
	150	0.01000	0.011386	0.001	−0.02591		0.04591
150	2	−0.29312	0.011386	0.000	−0.32903		−0.25722
	5	−0.18500	0.011386	0.000	−0.22091		−0.14909
	20	−0.05213	0.011386	0.002	−0.08803		−0.01622
	50	−0.01000	0.011386	0.001	−0.04591		0.02591

**Table 2:** Comparison of mean UVA according to time of exposure.

(I) time (min)	(J) time (min)	Mean Difference (I-J)	Std. Error	Sig.	95% Confidence Interval		
					Lower Bound	Upper Bound	
1	5	0.00460	0.010184	1.000	−0.02521		0.03441
30		0.00380	0.010184	1.000	−0.02601		0.03361
60		0.00220	0.010184	1.000	−0.02761		0.03201
5	1	−0.00460	0.010184	1.000	−0.03441		0.02521
30		0.00080	0.010184	1.000	−0.03061		0.02901
60		−0.00240	0.010184	1.000	−0.03221		0.02741
30	1	−0.00380	0.010184	1.000	−0.03361		0.02601
5		0.00080	0.010184	1.000	−0.02901		0.03061
60		−0.00160	0.010184	1.000	−0.03141		0.02821
60	1	−0.00220	0.010184	1.000	−0.03201		0.02761
5		−0.00240	0.010184	1.000	−0.02741		0.03221
30		−0.00160	0.010184	1.000	−0.02821		0.03141

Measurements are significant at 2, 5, 20, 50, 150 cm (P < 0.05). According to Table 2, the pairwise comparison of averages of UVA values at different times indicates no significant relationship between the mean of measurements at different times with the mean of measurements at all times (P > 0.05). Table 3, Table 4, respectively, show the results of univariate ANOVA of UVC measurement based on distance and time. There was no significant relationship for UV measurements at 5, 50, 150 cm (P > 0.05). Table 4, the pairwise comparison of UVC at different times indicates that there is no significant relationship between the mean of measured values at different times with the mean of the measurements in other times. The maximum and the minimum UVA are 0.321 and 0.006 W/m2, respectively, which are below the predefined limits. The highest and the lowest values of UVC radiation emitted from linear fluorescent lamps were 0.049 and 0.002 at 150 cm (Table 5, Table 6).The high UVC value is attributed to the defect in bulbs’ inner phosphor coating. To compare the UVA radiation intensity, the ANOVA was used. The results showed a significant relationship at 2, 5, 20, 50,150 cm (P < 0.05) (Table 1). There is no significant relationship at other cases (P > 0.05).

**Table 3:** Comparison of mean UVC emitted from the linear fluorescent lamps according to the distance.

(I) Distance (m)	(J) Distance (m)	Mean Difference (I-J)	Std. Error	Sig.	95% Confidence Interval	
					Lower Bound	Upper Bound
2	5	0.01475	0.003939	0.013	0.0233	0.02717
	20	0.02487	0.003939	0.000	0.01246	0.03729
	50	0.02913	0.003939	0.000	0.01671	0.04154
	150	0.03237	0.003939	0.000	0.01996	0.04479
5	2	−0.01475	0.003939	0.013	−0.02717	−0.00233
	20	0.01012	0.003939	0.182	−0.00229	0.02254
	50	0.01438	0.003939	0.016	0.00196	0.02679
	150	0.01763	0.003939	0.002	0.00521	0.03004
20	2	−0.02487	0.003939	0.000	−0.03729	−0.01246
	5	−0.01012	0.003939	0.182	−0.02254	−0.00229
	50	0.00425	0.003939	1.000	−0.00817	0.01667
	150	0.00750	0.003939	0.714	−0.00492	0.01992
50	2	−0.02913	0.003939	0.000	−0.04154	−0.01671
	5	−0.01438	0.003939	0.016	−0.02679	−0.00196
	20	−0.00425	0.003939	1.000	−0.01667	0.00817
	150	0.00325	0.003939	1.000	−0.00917	0.01567
150	2	−0.03237	0.003939	0.000	−0.04479	−0.01996
	5	−0.01763	0.003939	0.002	−0.03004	−0.00521
	20	−0.00750	0.003939	0.714	−0.01992	0.00492
	50	−0.00325	0.003939	1.000	−0.01567	0.00917

**Table 4:** Comparison of mean UVC according to the time of exposure.

(I) time (min)	(J) time (min)	Mean Difference (I-J)	Std. Error	Sig.	95% Confidence Interval		
					Lower Bound	Upper Bound	
1	5	0.00170	0.003523	1.000	−0.00861		0.01201
	30	0.00140	0.003523	1.000	−0.00891		0.01171
	60	0.00150	0.003523	1.000	−0.00881		0.01181
5	1	−0.00170	0.003523	1.000	−0.01201		0.00861
	30	−0.00030	0.003523	1.000	−0.01061		0.01001
	60	−0.00020	0.003523	1.000	−0.01051		0.01011
30	1	−0.00140	0.003523	1.000	−0.01171		0.00891
	5	0.00030	0.003523	1.000	−0.01001		0.01061
	60	0.00010	0.003523	1.000	−0.01021		0.01041
60	1	−0.00150	0.003523	1.000	−0.01181		0.00881
	5	0.00020	0.003523	1.000	−0.01011		0.01051
	30	−0.00010	0.003523	1.000	−0.01041		0.01021

**Table 5:** The measured values of UVA emitted from the linear fluorescent lamps.

**N**	40
**Mean**	0.11455
**Std. Deviation**	0.116296
**Range**	0.315
**Minimum**	0.006
**Maximum**	0.321
**Sum**	4.582

**Table 6:** The measured values of UVC emitted from linear fluorescent lamps.

**N**	40
**Mean**	0.01455
**Std. Deviation**	0.013204
**Range**	0.047
**Minimum**	0.002
**Maximum**	0.049
**Sum**	0.566

## Materials and methods

The study was performed on 40 linear fluorescent lamps in Iran. First, the light intensity of lamps was measured by the Luxmeter (Hagner, Model: EC1). If the difference of light intensity was more than 5% of other lamps, it would be excluded from the study. The watt range of selected lamps was between 11 and 40 W [[1], [2], [3], [4], [5], [6], [7]].

### Measurement of ultraviolet radiations

Before starting the measurements, all the lights were turned off, and then the UV radiations emitted from the ground was measured using a UV meter. The lamps that were to be measured were turned on for 10 min. The device was calibrated before any measurement to ensure the proper functioning of that. The ultraviolet radiation was measured when the lamps were turned on for the first time. The UV radiations emitted from the lamps were measured using an ultraviolet meter (Hanger model: S4) that consists of a sensor for UVA and UVC. The wavelength range (nm) of UVA and UVC is (400–300) and (100–280), respectively. Data analysis was carried out by SPSS version 18. To determine the type of statistical test, the data distribution was checked out by Shapiro Wilk test. The distribution of data was normal, so ANOVA was used to analyze the data.

## Conclusion

The results proved that the intensity of UVA emitted from the lamps was less than the permissible limit at all distances. However, this value is higher than the permissible limit for UVC at 150 cm.

## Conflict of interest

The authors of this article declare that they have no conflict of interests.
